# Genome sequence data of the strongly antagonistic yeast *Pichia kluyveri* isolate APC 11.10 B as a foundation for analysing biocontrol mechanisms

**DOI:** 10.1016/j.dib.2023.109394

**Published:** 2023-07-09

**Authors:** Lukas Nägeli, Martin Schuler, Tina Segessemann, Daniel Frei, Jürg E. Frey, Kenneth H. Wolfe, Christian H. Ahrens, Florian M. Freimoser

**Affiliations:** a Agroscope, Research Division Plant Protection, Route de Duillier 60, 1260 Nyon 1, Switzerland; b Agroscope, Competence Division Method Development and Analytics, Reckenholzstrasse 190, 8046 Zürich, Switzerland; c Conway Institute, University College Dublin, Dublin 4, Ireland; d SIB, Swiss Institute of Bioinformatics, Reckenholzstrasse 190, 8046 Zürich, Switzerland

**Keywords:** Antagonism, Biocontrol, Genome assembly and annotation, Mechanism, Plant protection, Yeast

## Abstract

*Pichia kluyveri* strain APC 11.10 B was isolated from apple bark in Switzerland and exhibited strong antagonistic activity against plant pathogenic fungi *in vitro* (e.g., *Botrytis, Fusarium* or *Monilinia* isolates). In order to identify the mechanisms underlying this antagonism, we have sequenced the genome of this isolate by long- and short-read sequencing technologies. The sequence data were *de novo* assembled into nine scaffolds and a fully resolved circularized mitogenome. The total genome size was 10.9 Mbp and 7451 potential open reading frames (ORFs) and 202 tRNA genes were predicted. In comparison to two *P. kluyveri* genomes deposited at the NCBI (of strains X31-10 and CBA6002), the APC 11.10 B strain seemed to represent a hybrid because backmapping of sequencing reads resulted in a high rate of heterozygous and structural variants in the nuclear genome (this was not observed for the mitochondrial genome). The *P. kluyveri* (APC 11.10 B) draft genome represents a first step and resource for genome mining, comparative and functional genomics (e.g., identifying the biocontrol mode of action), and evolutionary studies. Since the genus *Pichia* comprises many biotechnologically relevant yeasts, the genome data may be used in a variety of fields and disciplines.

**Specifications Table**

**Table:** 

Subject	Agricultural Microbiology
Specific subject area	Genome analysis of a yeast that strongly antagonises fungal plant pathogens.
Type of data	Draft genome sequence data, genome annotation, table and figure
How data were acquired	Genomic DNA sequencing by Oxford Nanopore Technologies (ONT), PacBio and Illumina MiSeq platforms, *de novo* assembly
Data format	Raw data: annotated draft genome assembly Secondary data: table of annotated genes, the encoding proteins, and functional prediction
Description of data collection	Genomic DNA was extracted from a pure culture of *P. kluyveri* (APC 11.10 B) using a phenol/chloroform protocol. **Sequencing:** Oxford Nanopore Technologies (ONT), PacBio, Illumina MiSeq **Assembly:** filtering using length cut-offs, *de novo* assembly of PacBio reads, scaffolding with long ONT reads, reference-based assembly of the mitogenome. **Annotation:** Yeast Genome Annotation Pipeline (YGAP) and KEGG Orthologs assignment with KofamKOALA.
Data source location	*P. kluyveri* (APC 11.10 B) was isolated from the bark of an apple tree that was collected in spring 2014 near Feldbach (47.239529 °N, 8.786822 °E, 415 m.a.s.l.), Switzerland. The strain is available at the Culture Collection of Switzerland under CCOS982.
Data accessibility	The genome is deposited at NCBI's Genbank under the BioProject PRJNA964584 and the accession numbers CP125793-CP125802 (https://www.ncbi.nlm.nih.gov/assembly/GCA_030062975.1). The raw sequencing data and genome annotation files are available as supplementary data at https://dataverse.harvard.edu/dataverse/Pichia_kluyveri.
Related research article	Hilber-Bodmer, M., Schmid, M., Ahrens, C.H., Freimoser, F.M., 2017. Competition assays and physiological experiments of soil and phyllosphere yeasts identify *Candida subhashii* as a novel antagonist of filamentous fungi. BMC Microbiol. 17, 4. https://doi.org/10.1186/s12866-016-0908-z

## Value of the Data

• The genome of *P. kluyveri* (APC 11.10 B) can be used as the basis for genome mining, comparative and functional genomics (e.g., elucidating the biocontrol mechanisms employed by this yeast), and evolutionary studies..
• The genome data can serve as a foundation for studying microbial interactions at the molecular level, developing new and improved biocontrol applications, or biotechnological applications.
• The genome may provide a valuable resource for biocontrol researchers, biologist, microbiologists, mycologists, bioinformaticians, or even biotechnologists.

## 1 Objetive

*Pichia kluyveri* (APC 11.10 B; CCoS982) was identified as a strongly antagonistic yeast [1]. As a foundation for elucidating the biocontrol mechanism employed by this yeast, the genome was sequenced, assembled and annotated. The genome sequence is a prerequisite and foundation for identifying potential biocontrol genes, performing transcriptome analyses, or identifying proteins and peptides.

## 2 Data Description

*Pichia kluyveri* (APC 11.10 B; CCoS982) was isolated from the bark of an untreated apple tree that was collected in spring 2014 near Feldbach (47.239529 °N, 8.786822 °E, 415 m.a.s.l.), in Switzerland. The strain was identified based on the ITS sequence and searching the UNITE database as the species hypothesis SH1527625.08FU, which corresponds to *Pichia kluyveri* (Bedford ex Kudryavtsev) [[1], [2], [3], [4]]. The isolate was one of the most strongly antagonistic yeasts against a range of saprophytic and plant pathogenic filamentous fungi (e.g., *Botrytis, Fusarium*, and *Monilinia* strains) [1]. It was thus the goal of this genome-sequencing project to generate the foundation for identifying the biocontrol mechanisms of *P. kluyveri* (APC 11.10 B).

The *P. kluyveri* APC 11.10 B genome was sequenced using Oxford Nanopore Technologies (ONT) and PacBio long-read sequencing, as well as Illumina short reads. All sequencing raw data (bam, fastq, and fasta files), as well as annotation files (plain text files of the amino acid sequences encoded by the predicted genes) are available at the Harvard dataverse for this genome (https://dataverse.harvard.edu/dataverse/Pichia_kluyveri). Integration of ONT, PacBio and Illumina reads, together with extensive polishing and manual curation (see section below), resulted in a final assembly of nine scaffolds and a mitogenome (total genome size 10’909’748 bp, including a circular mitogenome of 38’909 bp) with an average GC content of 28.3% (Table 1 and Fig. 1, circle 1 and 2). The read coverage of the assembled scaffolds 1-9 was between 103-111x except for scaffold 5 at 142x. The mitogenome had a coverage of 365x. The N50 was 1164.4 kbp and the largest scaffold was 2.1 Mbp. A total of 6.16% of the genome was identified as repeats of which putative telomeric ends with the sequence motif `CTATACCCCCCCTGCGACCTACTTCA` were found on scaffolds 1, 2, 6 and 9 (Figure 1, circle 6). Although, the same motif was not found in two other published *Pichia* assemblies, it may be a novel, fungal telomere motif not previously characterized in eukaryotes. The motif was found independently in assemblies generated for all three sequencing libraries by using the Tandem Repeats Finder tool [5].

**Table 1:** Statistics of the final, assembled scaffolds. Scaffolds with putative telomeric sequences are in italic.

Scaffold	Accession	Coverage	GC%	Length (bp)
*1*	*CP125793*	*103.1*	*27.71*	*2’105’441*
*2*	*CP125794*	*103.0*	*28.45*	*1’963’561*
3	**CP125795**	111.7	28.48	1’643’796
4	**CP125796**	106.8	28.08	1’310’187
5	**CP125797**	**142.0**	29.25	1’009’114
*6*	*CP125798*	*107.3*	*28.75*	*1’000’076*
7	**CP125799**	111.3	28.56	906’913
8	**CP125800**	111.1	28.41	653’620
*9*	*CP125801*	*108.9*	*28.34*	*278’131*
mtDNA	**CP125802**	365.2	22.72	38’909

**Figure:**
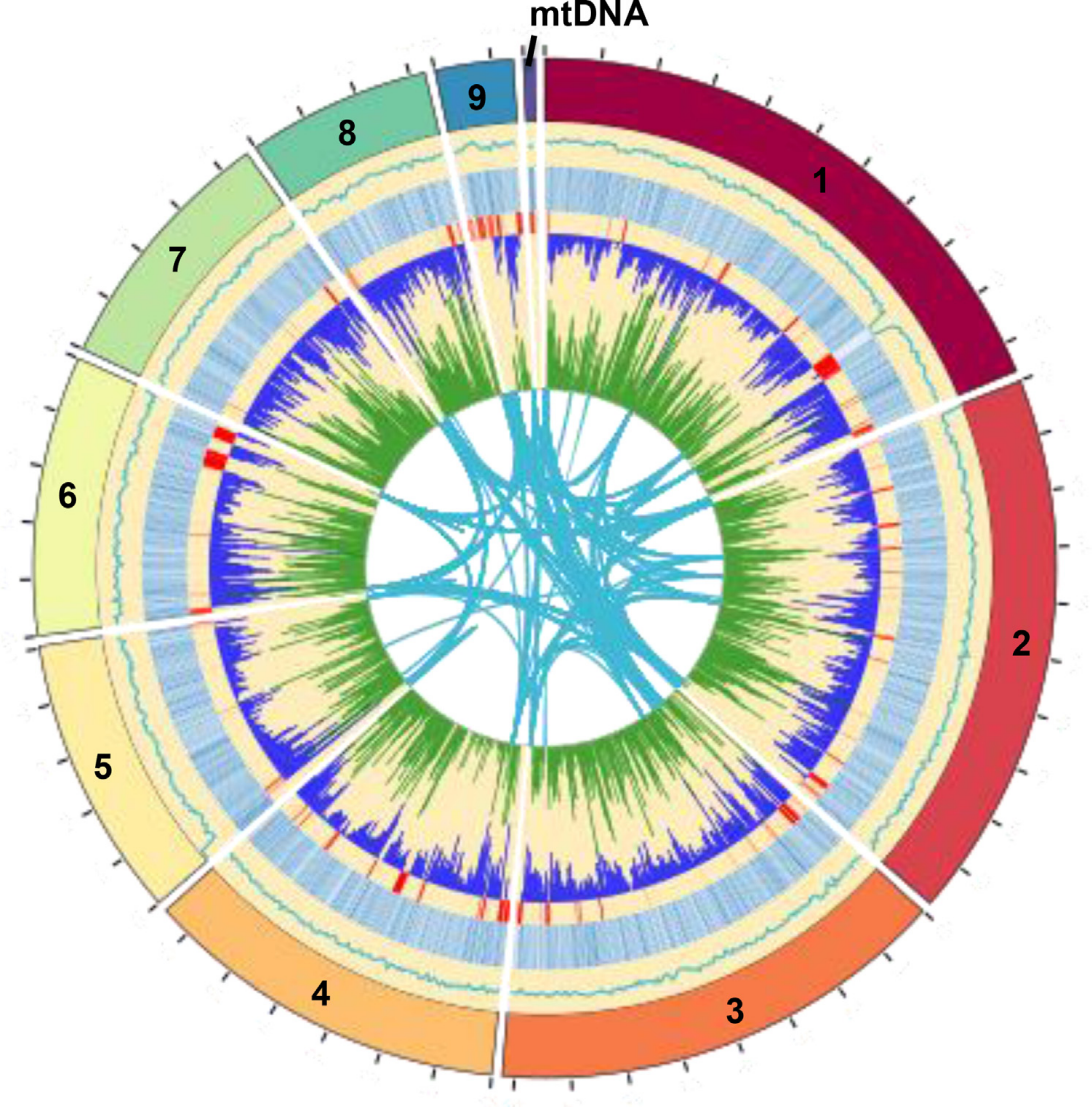
**Fig. 1:** BioCircos Plot of the *Pichia kluyveri* APC 11.10 B genome. Circle 1 (outermost): Scaffolds. ticksize = 200 kilo bases. Circle 2: relative GC content. Circle 3: ORF density where dark blue indicates higher ORF density. Circle 4: Gaps in the alignment to CBA6002 and X31-10. Circle 5: SNP density for CBA6002 (green) and X31-10 (blue). Circle 6: Duplications lager than 500 base pairs with at least 95% identity, an e-value smaller than 1x10^-10^ and a bitscore larger than 2000.

By using the Yeast Genome Annotation Pipeline (YGAP) and manual curation (identification of ORFs encoding for peptides of at least 100 amino acids in length), 7451 potential ORFs were annotated in the *P. kluyveri* APC 11.10 B genome (distribution shown in Figure 1, circle 3). In total, 2974 significant matches were detected by a KofamKOALA KEGG Orthology analysis (2900 predicted ORFs had significant matches to 2573 different KEGG orthology identifier (KO terms). Of all predicted genes, 5892 were characterised with at least one KO term (also counting matches below threshold), indicating that many ORFs had no prediction. The KofamKOALA KEGG Orthology analysis predicted complete pathways for C5 isoprenoid and mevalonate (M00095) as well C10-C20 isoprenoid (M00367) biosynthesis. The fungal antiSMASH v.6.0 tool [6] did not identify any potential secondary metabolite clusters. Genes with significant matches to the mating-type (MAT) locus transcription factor genes MATalpha1 and MATalpha2 of other *Pichia* species were found, located between the genes SLA2 and TGL1 as in *Pichia kudriavzevii* [7], which suggests that *P. kluyveri* APC 11.10 B is a mating type alpha strain. No significant matches to MATa1 or MATa2 genes were found. The publicly available genomes of two other *P. kluyveri* isolates (CBA6002 and X31-10, accession numbers JAGUCV010000000 and QEFR00000000, respectively), were of comparable size (12’399’968 and 10’964’178 bp, respectively). A Phylogenetic and Molecular Evolution (PhaME) analysis [8] with the three available *P. kluyveri* genomes showed a 93.5% and 95.5% linear coverage for CBA6002 and X31-10, respectively. The CBA6002 genome had thus more single nucleotide polymorphisms (SNPs; 97 per 10 kilo bases) than the X31.-10 genome (65 per 10 kilo bases) (Figure 1, circle 5), indicating that the isolate APC 11.10 B is more closely related to the X31-10 isolate. There were only 175 gaps larger than 1 kilo base in both alignments together. The largest two were located at the end of scaffold 6 (Figure 1, circle 4).

## 3 Experimental Design, Materials and Methods

Genomic DNA was extracted using a phenol/chloroform extraction protocol. The ONT library was prepared using a 1D2 Sequencing Kit (SQK-LSK308) and sequenced on a FLO-MIN107 (R9.5) flow cell. PacBio sequencing was carried out on a Sequel machine. Size selection was performed using the BluePippin system. Two 2×300 bp Illumina paired end libraries were prepared using the Nextera XT DNA kit and sequenced on a MiSeq.

The assembly strategy and data characterizing the different steps are summarized in Supplementary Table 1. PacBio reads were filtered using specific length thresholds (1 Kbp, 5 Kbp, 6.5 Kbp) with seqkit [9]. ONT reads were quality/length filtered using filtlong [10] with the settings ‘–min_mean_q 92 –min_length 1000-20000’. An initial, qualitative meta-Flye assembly of the ONT reads was further filtered by mapping against the full set of assembled scaffolds using ‘bwa mem’ [11]. Two short-read Illumina libraries were quality filtered using fastp [12]. Assemblies were generated using different programs and post-processed as summarized (Supplementary Table 1). Three separate Flye [13] assemblies using different input libraries were generated (ID 1-3). These assemblies were further processed using pseudohaploid [14] to remove redundant scaffolds, creating a chimeric assembly. On these pseudohaploid scaffolds, the LongStitch [15] pipeline was applied to correct potentially misassembled regions and for scaffolding, using either the PacBio library or ONT library. This step introduced gaps (Ns), the number of which was comparable to other published assemblies. Since the ONT library resulted in better contiguity than with PacBio reads, the ONT corrected assembly was further polished using pilon [16] using short-reads until convergance (3-4 rounds). From these three, Flye-assembly `2` had the best trade-offs in terms of contiguity and completeness. Scaffolds shorter than the 38’909 bp long mitogenome were discarded, which resulted in 13 scaffolds with 9 gaps, a size of 11.1 Mbp and a BUSCO score of C:94.7%[S:94.0%,D:0.7%], F:1.0%,M:4.3%. Further assembly curation included taxonomic classification and coverage analysis using BlobTools [17,18].

When either long-reads or short-reads were mapped back to the assemblies, a high rate of heterozygous variants as well as larger structural variants were observed. This hints at the possibility of a hybrid yeast. The mitogenome was not affected by these observations, therefore indicating a monocolonal single isolate in all three sequencing libraries. Long-read backmapping resulted in a higher coverage for scaffold 5 as compared to the other scaffolds (with the exception of the mitogenome, which has multiple copies per cell). This may indicate an assembly-error, collapsed regions, aneuploidy or other repeat-related problems that would need to be further investigated. A coverage analysis based on long-read mapping data is provided in Table 1 among the GC-content and scaffold length.

Three scaffolds (10, 11 and 12) were discarded due to lower coverage (32x, 38x and 37x; a BLAST against the final assembly returned several hits with a query coverage of ∼50-70%).

PhaME analysis with the complete genomes was performed according to the developer's instructions (see https://github.com/LANL-Bioinformatics/PhaME). Data consolidation and figures were generated with R version 4.2.2 (R [19]) mainly using the package BioCircos [20,21] (package version 0.3.4).

The *P. kluyveri* (APC 11.10 B) genome was annotated as previously described [22] by using the Yeast Genome Annotation Pipeline (YGAP) [23] and manual curation and correction in Artemis [24]. KEGG Orthologs (KOs; K numbers) were assigned to 7451 predicted proteins by KofamKOALA [21] and the KEGG Mapper Reconstruct tool was used to assign the KOs to pathway modules [15].

## Ethics statements

This work does not contain any studies with human or animal subjects.

## CRediT Author Statement

**Lukas Nägeli**: investigation, resources; **Martin Schuler, Tina Segessemann**: software, formal analysis; **Daniel Frei, Jürg E. Frey**: resources, supervision; **Kenneth H. Wolfe**: software, data curation, supervision; **Christian H. Ahrens**: conceptualization, software, supervision; **Florian M. Freimoser**: conceptualization, writing, supervision.

## Declaration of Competing Interest

The authors declare that they have no known competing financial interests or personal relationships, which have or could be perceived to have influenced the work reported in this article.

## Data Availability

Pichia kluyveri (APC 11.10 B) genome (Original data) (Harvard dataverse) Genome assembly (Original data) (NCBI).

## Appendix Supplementary materials

Image, application 1
