# Supplementary material for: Genome sequence data of the strongly antagonistic yeast Pichia kluyveri isolate APC 11.10 B as a foundation for analysing biocontrol mechanisms
Source: Data Brief. 2023 Jul 9;49:109394. doi: 10.1016/j.dib.2023.109394 (PMC10365976; doi:10.1016/j.dib.2023.109394)
Supplement: Supplementary file 1 [file mmc1.docx]

**Supplementary Table 1.** Overview of assemblies with used input sequencing libraries and software settings showing assembly-specific statistics. Post-processing was performed using the tools Pseudohap, Longstitch, Flye-polish and a short-read polisher Pilon. The assembly highlighted in yellow was used for further post-processing and to arrive at the final draft assembly. Sequencing coverage of the different libraries (shown in brackets, third column) was calculated with a genome size estimation of 11.1 Mbp.

| Assembly  (post-processing) | ID | Input and Settings, Comment | N50 (Kbp) | Contigs (Gaps) | Total Size (Mbp) | BUSCO  (*Saccharomycetes*, odb10, n2137) |
| --- | --- | --- | --- | --- | --- | --- |
| X31-10 (public *P. kluyveri*) | - | GenBank assembly accession: GCA_019096115.1 | 2’370.1 | 15 (3) | 10.96 | C:91.1%[S:90.2%,D:0.9%],F:3.5%,M:5.4% |
| Flye | 1 | >1 Kbp reads PB (148x),--pacbio-raw | 1’336 | 48 | 11.23 | C:94.2%[S:92.8%,D:1.4%],F:1.5%,M:4.3% |
|  | 2 | >5 Kbp reads PB (141x), >20 Kbp reads ONT (16x), --pacbio-raw | 1’282 | 35 | 11.33 | C:94.5%[S:92.9%,D:1.6%],F:1.1%,M:4.4% |
|  | 3 | >6.5 Kbp reads PB (136x), >20 Kbp reads ONT (16x), --pacbio-raw | 1’283 | 36 | 11.34 | C:94.2%[S:92.7%,D:1.5%],F:1.5%,M:4.3% |
| Flye (Pseudohap) | 1 | MIN_IDENTIY=90, MIN_LENGTH=1k, MIN_CONTAIN=93, MAX_CHAIN_GAP=2k | 1’336 | 42 | 11.1 | C:94.2%[S:93.4%,D:0.8%],F:1.5%,M:4.3% |
|  | 2 |  | 1’282 | 33 | 11.3 | C:94.5%[S:92.9%,D:1.6%],F:1.1%,M:4.4% |
|  | 3 |  | 1’283 | 34 | 11.3 | C:94.2%[S:92.7%,D:1.5%],F:1.5%,M:4.3% |
| Flye (Pseudohap, Longstitch PB) | 1 | >5 Kbp reads PB (141x) | 1’486 | 60 (12) | 11.1 | C:94.1%[S:93.4%,D:0.7%],F:1.5%,M:4.4% |
|  | 2 |  | 1’705 | 47 (12) | 11.3 | C:94.3%[S:92.7%,D:1.6%],F:1.1%,M:4.6% |
|  | 3 |  | 1’706 | 48 (12) | 11.3 | C:94.0%[S:92.5%,D:1.5%],F:1.5%,M:4.5% |
| Flye (Pseudohap, Longstitch ONT) | 1 | >1 Kbp reads ONT *(26.6x) | 1’483 | 35 (7) | 11.0 | C:93.9%[S:93.1%,D:0.8%],F:1.5%,M:4.6% |
|  | 2 |  | 1’644 | 35 (9) | 11.2 | C:94.4%[S:92.9%,D:1.5%],F:1.1%,M:4.5% |
|  | 3 |  | 1’312 | 31 (8) | 11.3 | C:94.2%[S:92.7%,D:1.5%],F:1.5%,M:4.3% |
| Flye (Pseudohap, Longstitch ONT, PILON, length filtering) | 1 | fastp trimming; -l 40 -c -g -x -p -M 30 -w 8 -5 -3 -b 100 -B 100,  pilon polish using trimmed reads (57.5x); --diploid --fix all Removal of contigs <38 Kbp | 1’483 | 12 (7) | 10.7 | C:94.1%[S:93.4%,D:0.7%],F:1.2%,M:4.7% |
|  | *2* |  | 1’644 | 13 (9) | 11.1 | C:94.7%[S:94.0%,D:0.7%],F:1.0%,M:4.3% |
|  | 3 |  | 1’312 | 15 (8) | 11.2 | C:94.5%[S:93.4%,D:1.1%],F:1.3%,M:4.2% |
| *Final draft assembly* | *2* | ***three low-coverage contigs discarded  (as compared to assembly highlighted in yellow above)*** | ***1’164*** | ***10 (9)*** | ***10.9*** | ***C:94.7%[S:94.0%,D:0.7%],F:1.0%,M:4.3%*** |
